# Quantal Glutamate Release Is Essential for Reliable Neuronal Encodings in Cerebral Networks

**DOI:** 10.1371/journal.pone.0025219

**Published:** 2011-09-20

**Authors:** Jiandong Yu, Hao Qian, Na Chen, Jin-Hui Wang

**Affiliations:** 1 State Key Lab for Brain and Cognitive Sciences, Institute of Biophysics, Chinese Academy of Sciences, Beijing, China; 2 Graduate School of Chinese Academy of Sciences, Beijing, China; Emory University, United States of America

## Abstract

**Background:**

The neurons and synapses work coordinately to program the brain codes of controlling cognition and behaviors. Spike patterns at the presynaptic neurons regulate synaptic transmission. The quantitative regulations of synapse dynamics in spike encoding at the postsynaptic neurons remain unclear.

**Methodology/Principal Findings:**

With dual whole-cell recordings at synapse-paired cells in mouse cortical slices, we have investigated the regulation of synapse dynamics to neuronal spike encoding at cerebral circuits assembled by pyramidal neurons and GABAergic ones. Our studies at unitary synapses show that postsynaptic responses are constant over time, such as glutamate receptor-channel currents at GABAergic neurons and glutamate transport currents at astrocytes, indicating quantal glutamate release. In terms of its physiological impact, our results demonstrate that the signals integrated from quantal glutamatergic synapses drive spike encoding at GABAergic neurons reliably, which in turn precisely set spike encoding at pyramidal neurons through feedback inhibition.

**Conclusion/Significance:**

Our studies provide the evidences for the quantal glutamate release to drive the spike encodings precisely in cortical circuits, which may be essential for programming the reliable codes in the brain to manage well-organized behaviors.

## Introduction

Brain functions are fulfilled by neural circuits, in which the synapses transmit the spike signals encoded at the neurons [1], [2], [3], [4], [5]. It is well known about that the patterns of presynaptic spikes regulate synaptic transmission [6], and induce a plasticity at the synapses and/or neurons [7], [8], [9], [10], [11], [12], [13], [14], [15]. Little is known about how synapse dynamics influences spike patterns at postsynaptic neurons, especially their precise encoding. A solution for this essential question is to investigate the quantitative correlations between synapse dynamics and neuronal encoding in brain networks.

Synapse dynamics is affected presynaptically by the probability of transmitter release, number of release sites and content of released transmitter [6], [16], [17]. It is not conclusive whether a fluctuated synapse dynamics in the CNS [18], [19], [20], [21] results from the change in transmitter release content or probability [22], [23], [24], [25], [26]. To address this issue, we estimated glutamate contents released from individual vesicles into the cleft of unitary synapses by inducing spikes in a pyramidal neuron and recording electrical signals from two postsynaptic sites, excitatory postsynaptic currents (uEPSC) at the GABAergic neurons and glutamate transport currents (uGTC) at the astrocytes in cortical slices.

**Figure 1:**
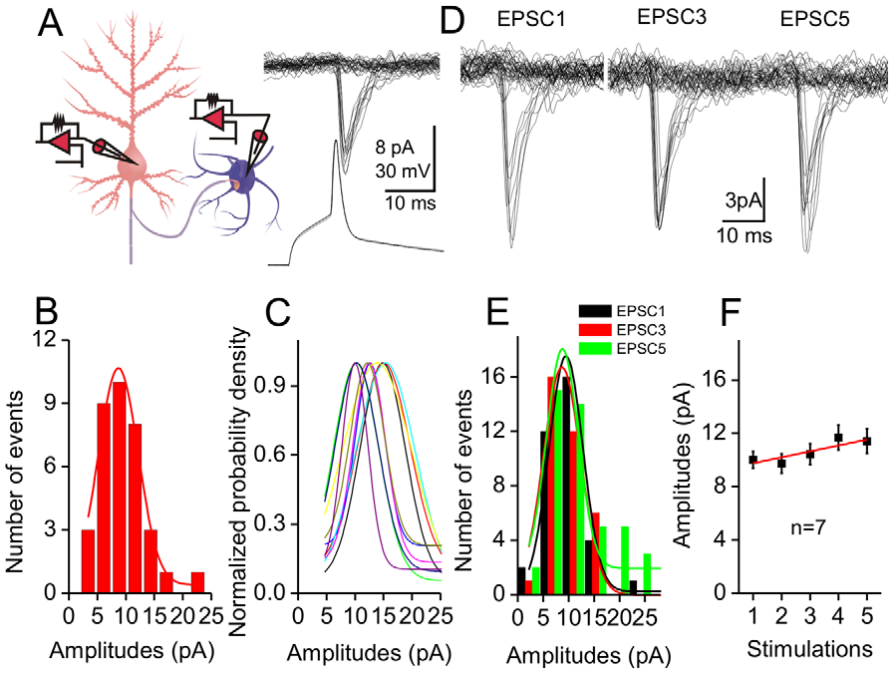
Glutamates released from synaptic vesicles appear constancy. **A**) Left panel shows a diagram for a pair-recording of uEPSCs at unitary synapses from a pyramidal to GABAergic neurons, which have a low release probability. Right panel shows the superimposed traces of uEPSCs (top) evoked by single spikes (bottom) at a synapse. **B**) The distribution of uEPSCs (non-failure portion of trails) appears single peak at this synapse, which is fitted based on Gaussian function.
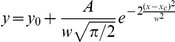
 **C**) shows the distributions of uEPSCs without failure portion from other synapses (n = 10). **D**) shows the superimposed traces of uEPSC1, 3 and 5 induced by sequential spikes at a synapse. **E**) The distributions of uEPSC1, 3 and 5 without failure portion appear a single peak at this synapse. **F**) shows a plot of uEPSC1∼5 amplitudes averaged from other synapses (n = 7, p>0.05 for the comparison of these uEPSCs).

Although synaptic patterns modulated by postsynaptic mechanisms influence neuronal encodings [21], it remains unclear about how presynaptic factors by setting synaptic activity patterns regulate signal integrations and spike encodings at postsynaptic neurons. In the neural circuits consisting of pyramidal and GABAergic neurons (Fig. 1A), how do the synapses on GABAergic cells drive their spike encodings and in turn regulate encodings at pyramidal cells? We investigated these questions with a particular attention to a role of glutamate release patterns in neuronal encodings.

## Results

### Glutamatergic synapses on GABAergic neurons release transmitters in quantal units

To estimate glutamate contents released from individual synaptic vesicles by measuring uEPSCs, we should rule out the effects of releasing vesicles from multiple sites on synaptic strength. A strategy in our study was the analysis of glutamatergic synapses with low release probability (*p*<0.25). As the synchronous incidence of independent events equals to a multiplication of their probabilities, a low *p* reduces the chance of releasing two vesicles synchronously and the effect of release-sites on synaptic strength. Moreover, our experiments were conducted without postsynaptic manipulation, i.e., receptor responsiveness was fixed. Under these conditions, uEPSCs likely signify glutamate contents released from single vesicles. uEPSCs were recorded at unitary glutamatergic synapses from a pyramidal cell to a GABAergic cell (Fig. 1A) in mouse cortical slices, where GABAergic neurons were genetically labeled by GFP (Methods).

The properties of uEPSCs recorded from low *p* glutamatergic synapses are illustrated in Fig. 1. uEPSCs (top panel) evoked by single spikes (bottom) at a synapse are less variable in amplitudes (1A). Excluding synaptic failure, a distribution of uEPSCs at this synapse shows a single peak (a simulative line in 1B), which is also observed at other synapses (one of color lines for a synapse in Fig 1C, n = 10). We then analyzed uEPSCs evoked by sequential spikes. uEPSC1, 3 and 5 at a synapse are similar in amplitudes (Fig. 1D). The distributions of their peaks overlap (Fig. 1E). uEPSC1∼5 values averaged without failure portion are not statistically different (Fig. 1F, n = 7). The contents of released glutamate are constancy at these unitary synapses.

**Figure 2:**
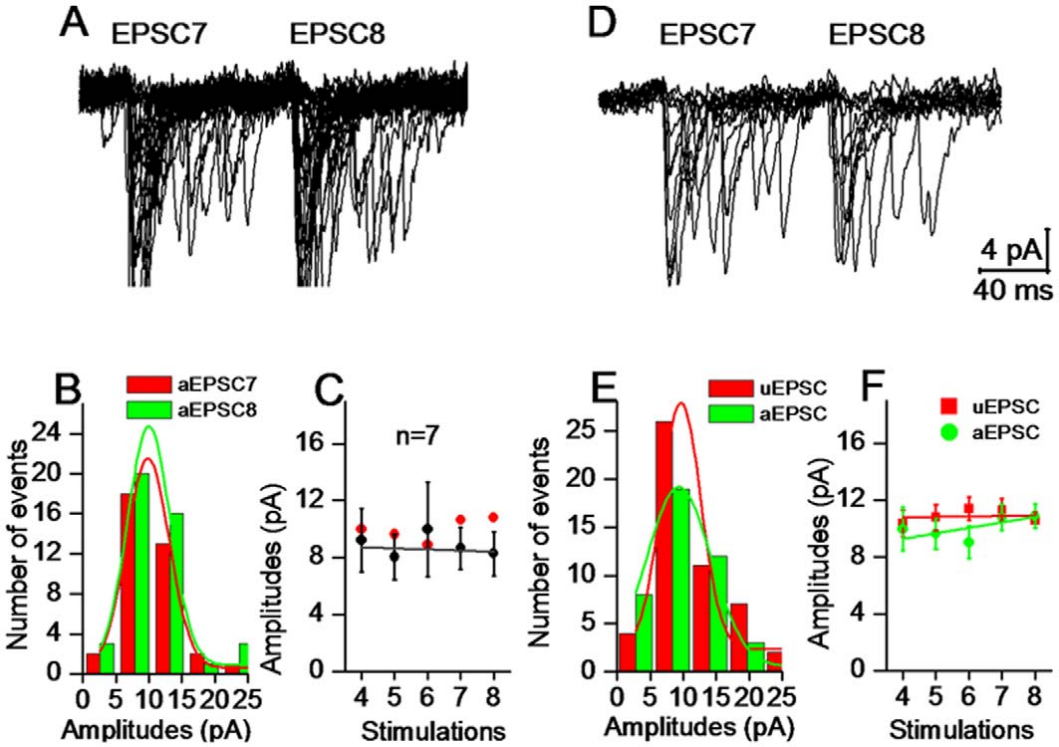
Glutamates released from synaptic vesicles by sequential spikes are constant. Asynchronous EPSCs following uEPSCs were recorded at glutamatergic unitary synapses from pyramidal to GABA neurons. **A**) shows the superimposed waveforms of aEPSCs after uEPSC7∼8 (out of the scale) at a synapse. **B**) The distributions of aEPSC7∼8 at this synapse appear a single peak and overlap. **C**) shows the plot of aEPSC4∼8 averaged from other synapses (n = 7, p>0.05 for the comparison of these aEPSCs). **D**) shows aEPSCs after uEPSC7∼8, which have similar amplitudes at a unitary synapse. **E**) The distributions of uEPSC8 versus aEPSC8 at this synapse are overlap. **F**) shows uEPSC4∼8 (black symbols) and aEPSC4∼8 (reds) averaged from this synapse (p>0.05 for the comparison of uEPSC4∼8 and aEPSC4∼8).

In addition, we examined the constant contents of released glutamate by analyzing asynchronous EPSCs. aEPSCs are associated with spike-evoked uEPSCs and usually expressed after spike-evoked uEPSC4 in our study, indicating that they are produced from an unitary synapse and aEPSCs depend on high release probability. aEPSCs were presumably evoked by the transmitters released from single vesicles [27], [28]. Fig. 2A illustrates aEPSC7∼8 following their corresponding uEPSCs at a unitary synapse. The distributions of aEPSC7 and aEPSC8 at this synapse overlap in their peaks (Fig. 2B). The averaged values for aEPSC4∼8 at this synapse (red symbols) and other synapses (blacks in Fig. 2C, n = 7) are not statistically different. It is noteworthy that we observed that the amplitudes of uEPSCs and aEPSCs from few synapses were matched well, indicating a single release site. Fig. 2D illustrates uEPSC7∼8 and their associated aEPSC7∼8 at a unitary synapse. The distributions of uEPSCs and aEPSCs at this synapse overlap in their amplitudes (Fig. 2E). The averaged values for uEPSC4∼8 (black symbols) and aEPSC4∼8 (reds in Fig. 2F) at this synapse and others (n = 2) are not statistically different (P>0.05). Thus, the glutamates released from single vesicles in evoked and asynchronous manners are constancy.

### Even glutamates are released to synaptic cleft from the terminals of pyramidal neurons

**Figure 3:**
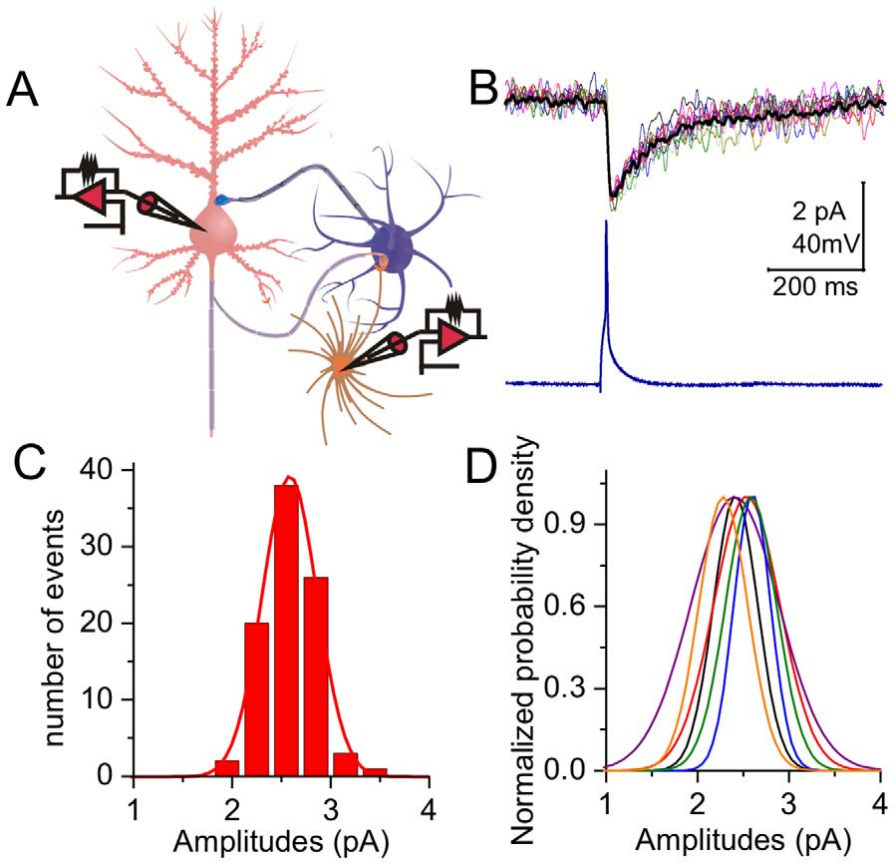
Glutamates released from presynaptic vesicles into synaptic cleft are constancy. **A**) Unitary glutamate transport currents (uGTCs) were recorded at unitary synapses from a pyramidal neuron to astrocyte. **B**) Single spikes (bottom panel) at a pyramidal neuron evoke uGTCs (superimposed waveforms in top panel) at an astrocyte. **C**) The distribution of uGTCs at this synapse appears a single peak. **D**) illustrates the distributions of uGTCs from other synapses (n = 6).

**Figure 4:**
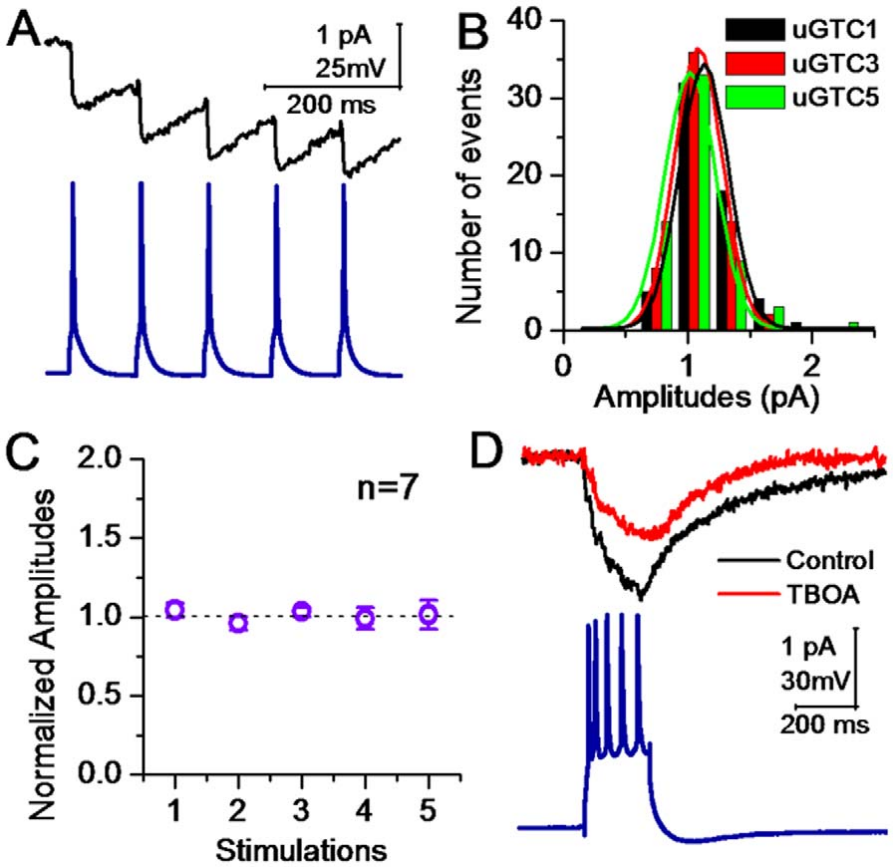
Glutamates released from synaptic vesicles by sequential spikes into synaptic cleft are constancy. uGTCs were recorded at unitary synapses from a pyramidal neuron to an astrocytes. **A**) uGTC1∼5 are induced by five sequential spikes. **B**) shows the distributions of uGTC1∼5 at this synapse. **C**) illustrates uGTC1∼5 averaged from other synapses (n = 7). **D**) uGTC1∼5 are blocked by TBOA, an antagonist of glutamate transporter.

Glutamates released from presynaptic vesicles act onto all of the postsynaptic identities, such as the spines of neurons and the processes of astrocytes around the synapses (synapse ensheathment). We estimated release contents by recording glutamate transport currents (GTC) at astrocytes [29] to examine whether glutamates released from single synaptic vesicles are constancy. In pair-recordings from a pyramidal neuron to an astrocyte (Fig. 3A), uGTCs (top panel in Fig. 3B) were evoked by presynaptic spikes (bottom). The distributions of uGTC amplitudes appear a single peak at this synapse (Fig. 3C) and others (one of color lines for a synapse in Fig 3D, n = 6). We also analyzed the distributions of uGTCs evoked by sequential spikes. Fig. 4A shows uGTC1∼5 (top panel) evoked by their corresponding spikes (bottom panel). The distributions of uGTC1, 3 and 5 amplitudes at this synapse overlap (Fig. 4B). The average values for uGTC1∼5 are not statistically different (Fig. 4C, n = 7). These uGTCs are partially blocked by DL-threo-β- Benzyloxyaspartate (TBOA, Fig. 4D), an antagonist of glutamate transporters [30], [31]. A constancy in uGTCs implies even glutamates released from presynaptic vesicles to synaptic cleft.

Our studies at cortical unitary synapses demonstrate that uEPSCs and aEPSCs at the GABAergic neurons or uGTCs at the astrocytes are constant in their amplitudes. The glutamates released among individual synaptic vesicles appear constant in quanta. We subsequently investigated the physiological significance of quantal glutamate release, whether synaptic patterns with constant quanta, i.e., a stable synaptic transmission, are benefit to the precise encoding of the neurons.

### Quantal glutamate release is beneficial to precise spike encoding in network neurons

Many synapses are convergent onto a neuron, and their integrated signals drive this cell encoding digital spikes. The transmission patterns of these synapses influence signal integrations and in turn spike encoding at postsynaptic neurons [21]. Compared with variable releases, do the synapses in quantal release drive spike encodings more reliably at postsynaptic neurons? In GABAergic neurons (Figs. 1∼2), how do the integrated signals regulate their spike encoding and then influence their downstream neurons via negative feedback?

**Figure 5:**
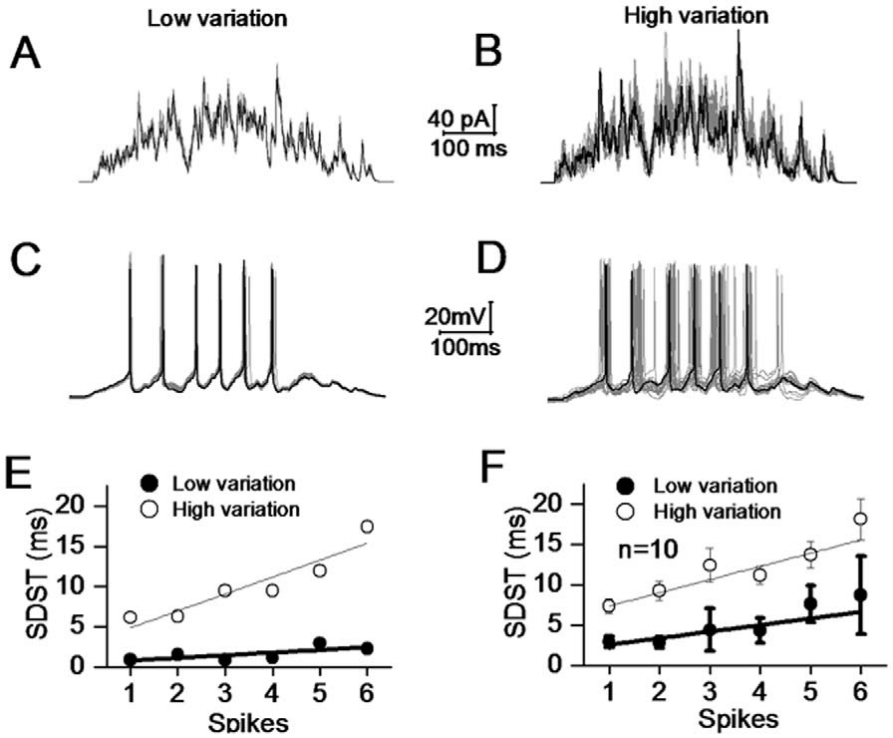
Signals integrated from excitatory synapses in constant quantum drive GABAergic neurons to precisely encoding action potentials. Left panels illustrate spike patterns driven by the signals integrated from synapses in constant quantum, and right panels are the results from synapses in variable quanta. A) presents the superimposed waveforms integrated from unitary synapses in constant quanta (low variation). B) shows the superimposed waveforms integrated from unitary synapses in variable quanta (high variation). C) Spike patterns driven by signals integrated from synapses in constant quanta appear precise and reliable. D) Spike patterns driven by signals from variable quantal synapses appear not precise. E) shows analytic data for standard deviation of spike timing (SDST) at this neuron, where the filled symbols are the results from synapses in constant quantal size and open symbols are from synapses in variable quanta. F) shows the SDST of spikes 1∼6 under two conditions (n = 10).

We examined the precise states of spike encodings in GABAergic neurons driven by the synapses in constant vs. variable quanta. As it is impossible to measure signal integrations from many synaptic inputs onto a neuron by simultaneously recording them, the integrations were done by mathematical approach [21]; Methods). In the math integration, we took the following factors into account, such as the glutamate contents released from single vesicles, the probability of transmitter release, the number of release sites, the sensitivity of postsynaptic glutamate receptors, the number of synapses onto a GABAergic neuron, and the input intervals from different presynaptic neurons. The synapses in constant quantal size vs. variable one are defined as their standard deviation of averaged uEPSCs to be 2.1 and 8.4 pA, respectively, based on the data in Figs. 1∼2. Other parameters for the synapses are given in Method (also [21]). The signals integrated from these unitary synapses are showed in top panels of Fig. 5 (left waves in 5A from constant quantal size, and right in 5B from variable size).

The integrated signals were injected into GABAergic cells that were genetically labeled by GFP to examine their precise states of spike encoding in Fig. 5 (n = 10). Spike patterns (Fig. 5C) driven by the signals from the synapses in constant quanta (5A) are precise and reliable, compared with those (Fig. 5D) driven by the signals from variable quanta (5B). The standard deviation of spike timing (SDST) at this neuron is showed in Fig. 5E, in which black symbols are data from the synapses in constant quanta and open symbols are from those in variable quanta. Fig. 5F shows SDST of spikes 1∼6 under these two conditions. Values for SDST_1_ to SDST_6_ are 2.94±0.66, 2.88±0.67, 4.4±2.64, 4.33±1.55, 7.65±2.27 and 8.74±4.8 ms from the signals in constant quanta (black symbols); and the values are 7.4±0.9, 9.3±1.2, 12.45±2.1, 11.2±1.13, 13.77±1.6 and 18.16±2.55 ms in variable quanta (open symbols). SDST values for corresponding spikes between the two conditions are statistically different (p<0.01). Spike patterns driven by the synapses with constant quanta on cortical GABAergic neurons are precise and reliable.

**Figure 6:**
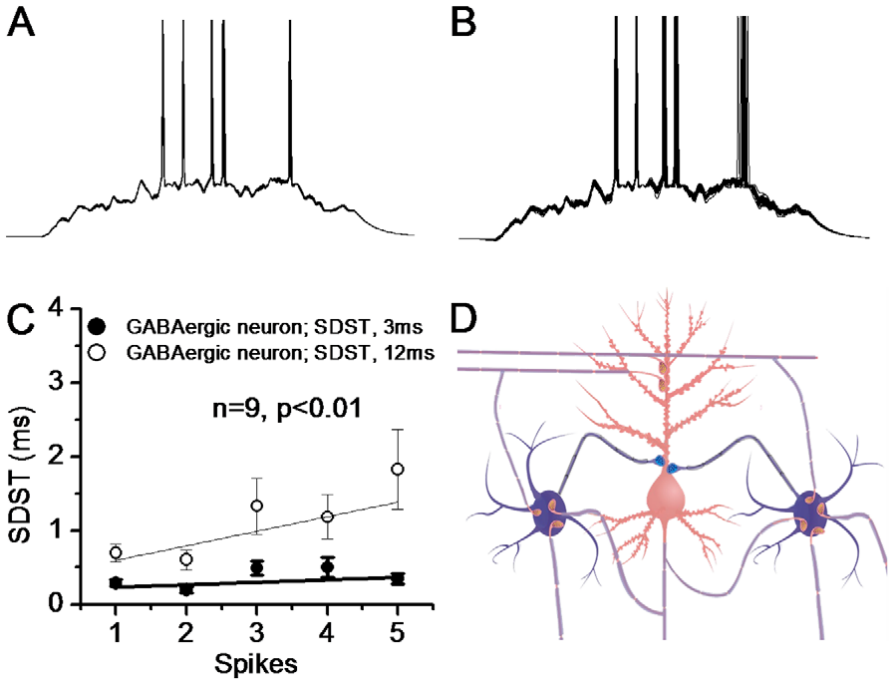
Pyramidal neurons driven by the signals integrated from excitatory and inhibitory synapses encode precise action potentials when spike patterns at GABAergic neurons are precise. A) Spike patterns on a pyramidal neuron driven by signals from precise GABAergic cells' spiking appear precise and reliable. B) Spikes driven by the signals from non-precise GABAergic cells' spiking are less precise. C) shows analytic data for standard deviation of spike timing (SDST) for spikes 1∼5 at pyramidal neurons under these two conditions (n = 9). Black symbols are its spike timing driven by precise GABA cell spiking (SDST, 3–6 ms), and open symbols are data from non-precise GABAergic cell spiking (SDST, 8–12 ms). D) shows the cerebral circuits that consist of pyramidal and GABAergic neurons. GABAergic neurons receive excitatory inputs from pyramidal neurons. Pyramidal neurons receive excitatory and inhibitory inputs that are from GABAergic feedback and feedforward routes.

In cerebral cortex, the neuronal circuits consist of pyramidal and GABAergic neurons, and they interact each other through the synapses (Fig. 1A). Does the precise and reliable spike encoding at GABAergic neurons (Fig. 5) grant spike encoding at pyramidal neurons to be reliable via feedback inhibitions? Pyramidal neurons are activated by the signals integrated from excitatory synapses, and regulated by inhibitory synapses from GABAergic neurons (Fig. 6D). In the modeling, the spike patterns at GABAergic cells are classified into precise and non-precise. The precise state of neuronal encoding is presented as the standard deviation of spike timing. SDST values for GABAergic neurons driven by the synapses in constant quanta vs. variable one are 3∼6 and 8∼12 ms, respectively (Fig. 5). Other parameters for signal integrations from excitatory and inhibitory synapses are given in Methods (also [1]. Resting membrane potential and threshold potential of pyramidal neurons are −65±2 mV and 22±1.5 mV, respectively [32].

Fig. 6 illustrates the spike patterns of pyramidal neurons driven by the signals integrated from excitatory and inhibitory synapses, when the encodings of GABAergic cells are precise (left column) or non-precise (right). Spike patterns driven by precise GABA-neuron encodings appear precise and reliable (Fig. 6A), compared with those driven by non-precise GABA-cell encoding (Fig. 6B). Fig. 6C shows the averaged data for the SDST of spikes 1∼5 at pyramidal neurons, in which black symbols are under precise GABA-cell encoding (SDST, 3–6 ms) and open symbols are data from non-precise GABA-cell encoding (SDST, 8–12 ms). The values for SDST_1∼5_ are 0.28±0.046, 0.2±0.036, 0.49±0.1, 0.5±0.14 and 0.34±0.07 ms under GABA-cell precision (black symbols); and values are 0.69±0.12, 0.6±0.13, 1.32±0.4, 1.18±0.3 and 1.83±0.54 ms under GABA-cell non-precision (opens). SDST values for corresponding spikes under these two conditions are statistically different (p<0.01, n = 9). Spike encodings at the pyramidal neurons will be more precise when GABAergic neurons fire spikes precisely.

## Discussion

We studied how synaptic transmission patterns influence neuronal encodings at a typical circuit consisting of pyramidal and GABAergic neurons in the brain (Fig. 6D). The vesicles from pyramidal neurons release the quantal units of glutamates (Figs. 1∼4). The activities of quantal synapses drive postsynaptic GABAergic cells precisely encoding spikes (Fig. 5), which in turn makes pyramidal cells to encode precise spikes (Fig. 6). Therefore, the quantal release of glutamates at the CNS synapses is one of efficient ways to maintain the reliable signal encodings in neural circuits. Precise input signals to the CNS neurons award them encoding digital spikes reliably, similar to the memory retrieval and playback by a process that specific inputs induce precise outputs.

Our studies by analyzing uEPSCs at low release probability synapses, uEPSCs-aEPSCs at unitary synapses and uGTCs at astrocytes without postsynaptic manipulations create a common conclusion that the glutamates released from presynaptic vesicles are constant in quanta. This nature in releasing glutamates from the cortical synapses supports a concept of quantal release established at peripheral synapses [33]. Synaptic strength is regulated by release probability, release sites and quantal size. The quanta are influenced by vesicle contents, release patterns and postsynaptic receptor responses [16], [20], [34], [35]. As the synchronous incidence of independent events equals to the multiplication of their probabilities, a low probability reduces a chance of synchronously releasing two vesicles and an effect of release-sites on uEPSCs. Together this with no postsynaptic manipulation, the constancies in uEPSCs and uGTCs are likely caused by releasing quantal glutamates from synaptic vesicles. Whether glutamate quantity is consistently packed among vesicles or released from them remains to be examined.

It is still not conclusive whether the fluctuations of synaptic strength under basal condition and plasticity result from the variations in the probability or the contents of transmitter release [16], [18], [20], [23], [25], [26], [35]. One of solutions to this question is to estimate glutamate content released from individual vesicles into the cleft of unitary synapses, for which we evoked spikes in a pyramidal neuron and recorded the signals from two postsynaptic sites, uEPSCs at GABAergic cells and uGTCs at astrocytes. The constant glutamate contents (Figs. 1∼4) suggest that the fluctuations in synaptic strength result from the changes in transmitter release probability and release-site numbers.

The fluctuations of synaptic strength are also proposed to be due to variable transmitter release patterns, i.e., full-fusion and kiss-and-run [35], [36]. If two release patterns are present in unitary synapses we studied, the constancies in uEPSCs, aEPSCs and uGTCs (Figs. 1∼4) imply that these two patterns release the same amount of transmitters. On the other hand, if two release patterns discharge the different amounts of glutamates [35], [37], the constancy of releasing glutamates (Figs. 1∼4) indicates that there is only one type of release patterns at the synapses from pyramidal to GABAergic neurons. In case of precise spike encodings driven by the synapses with constant quanta (Fig. 5), one type of release patterns, either full-fusion or kiss-and-run, is expected to be present at glutamatergic synapses.

It is noteworthy that evoked uEPSCs are larger than asynchronous EPSC easily makes an impression that they are from different release sites. aEPSCs are associated with spike-evoked uEPSCs and usually expressed after spike 4 in our studies (Fig. 2), indicating that they are produced from an unitary synapse. As we known, the glutamates previously released may result in a desensitization of postsynaptic receptors, which attenuates subsequent synaptic responses [38], [39]. The lower amplitudes of aEPSCs, compared with uEPSCs, are due to a possibility that glutamate receptors are still in a period of desensitization caused by glutamates released for inducing uEPSCs. On the other hand, even though uEPSCs and aEPSCs are produced by glutamates from different release sites, a single and narrow peak in the distribution of their amplitudes (Figs. 1∼2) indicates that the release patterns from both of them are quantal in nature.

Although our studies did not indicate a variable quantal release of glutamates at the unitary synapses, we made a case to test the influence of variable quantal release on neuronal spike encoding. The timing precision of spike encoding is worse when driven by the currents integrated from variable quantal release, compared with those from quantal release (Fig. 5). If the time precision of neuronal encoding underlies the fidelity of brain codes for well-organized behaviors and memory storage, the variable release quanta and subsequent non-precise neuronal encoding may be related to the functional and psychological disorders in the brain. As the release quanta are controlled by the amount of transmitters in synaptic vesicles and the patterns of their release, the uneven glutamates in synaptic vesicles and the conversion of a release pattern into two states will lead to non-precise encodings in neuronal network and functional disorders in the brain.

GABAergic neurons receive glutamatergic synapses that release transmitters from vesicles in constant quanta (Figs. 1∼4), and influence the activities of their postsynaptic neurons via the feedback and feedforward ways. The constant glutamates released from the synapses onto GABAergic neurons drives their spike encoding precisely (Fig. 5). The precise encoding of GABAergic neurons facilitates reliable spike encoding at their postsynaptic pyramidal neurons (Fig. 6). Thus, GABAergic neurons located around excitatory neurons maintain the latter to precisely encode action potentials, in addition to inhibiting postsynaptic neurons and elevating their sensitivity to the inputs [1], [40]. Pyramidal neurons while releasing constant glutamates from synaptic vesicles onto their targets have set up the reliable encoding themselves, a self set-point in homeostasis. The precise encoding of action potentials may circulate among circuitry neurons in the brain. If this chain homeostasis is broken, non-precise encodings in brain networks lead to functional disorders.

Glutamatergic synapses in constant quanta drive neuronal encoding more precisely and reliably. If precise neuronal encodings are essential to control well-organized behaviors, it is important to maintain synaptic quantal release, i.e., even glutamates are packed in synaptic vesicles and released from vesicles in a fixed pattern (either kiss-and-run or full-fusion). Glutamate quantity in presynaptic vesicles is influenced by the functions of vesicle glutamate transports and the gradient of glutamates between inside and outside of vesicles [41], [42], [43]. Both processes are ATP-dependent, i.e., rely on cellular metabolisms. Therefore, metabolic disorders lead to non-quantal release from synaptic vesicles and subsequent instability in neuronal encoding. On the other hand, the glutamate release patterns may be regulated in a conversion between kiss-and-run and full-fusion. It remains to be tested how the conversion of release patterns is regulated by presynaptic signals.

The patterns of synaptic transmission are regulated by quantal sizes, release probability and release sites. The quantal size is affected by the release contents of transmitters and the responsiveness of postsynaptic receptors [16], [20], [35]. In terms of the influences of synaptic factors on neuronal encodings, we have studied the roles of postsynaptic glutamate receptors [21], [44] and presynaptic release quanta in regulating the precise encodings of action potentials in cortical neurons. How the release probability influences neuronal encoding is under the study.

## Materials and Methods

### Brain slices

The study and all experiments conducted were fully approved by the Institutional Animal Care Unit Committee (IACUC) in Administration Office of Laboratory Animals Beijing China (ID# B10831). Cortical slices (400 µm) were prepared from FVB-Tg(GadGFP)45704Swn/J mice whose GABAergic neurons express green fluorescent protein (GFP; Jackson Lab, USA; [45]. Mice in postnatal day15–25 were anesthetized by injecting chloral hydrate (300 mg/kg) and decapitated with a guillotine. The slices were cut with a Vibratome in the oxygenized (95% O_2_/5% CO_2_) artificial cerebrospinal fluid (ACSF mM: 124 NaCl, 3 KCl, 1.2 NaH_2_PO_4_, 26 NaHCO_3_, 0.5 CaCl_2_, 5 MgSO_4_, 10 dextrose and 5 HEPES; pH 7.35) at 4^o^C, and then were held in the normal oxygenated ACSF (mM: 124 NaCl, 3 KCl, 1.2 NaH_2_PO_4_, 26 NaHCO_3_, 2.4 CaCl_2_, 1.3 MgSO_4_, 10 dextrose and 5 HEPES; pH 7.35) 24°C for 1∼2 hours before the experiments. A slice was placed to a submersion chamber (Warner RC-26G) that was perfused with the normal oxygenated ACSF at 31°C for whole-cell recordings [15], [46], [47].

### A selection of pair-recorded cells

Brain cells in layer II–IV of sensorimotor cortex were selected for pair-recordings. In synapse-coupled neurons, principal neurons have a pyramidal-like soma and an apical dendrite, whereas GABAergic neurons appear a round soma with multiple processes under DIC optics and GFP imaging (excitation, 488 and emission 525) under fluorescent microscope (Nikon FN- 600). These two types of neurons show different responses to hyperpolarization and depolarization pulses [45], [47], [48]. In the pair-recordings between a pyramidal neuron and a glia cell, the astrocytes are located at axonal side of pyramidal cells, and their processes enclosed the synapses made from the axons of pyramidal neurons.

### Dual whole-cell recording and glutamate content measurement

Single or multiple spikes in the presynaptic pyramidal neurons were evoked by injecting depolarization pulses at 0.1 Hz. The pulse durations were 10 ms with an intensity to initiate single spikes in presynaptic neurons, which evokes the mono-peak responses of unitary synapses, such as excitatory postsynaptic currents (uEPSC) at the GABAergic cells or glutamate transport currents (uGTC) at the astrocytes. These methods have been proposed to measure glutamate quantity released from presynaptic terminal [21], [49].

A MultiClamp-700B amplifier (Axon Instrument, Inc. Foster CA, USA) in current-clamp model produced paired-depolarization pulses (inter-pulse intervals, 50∼100 ms) to evoke presynaptic spikes. Voltage-clamp was used to record uEPSCs at GABAergic cells (holding potential, −70 mV) or uGTCs at astrocytes (holding, −90 mV). Electrical signals were inputted into pClamp-9 (Axon Instrument, Inc) for data acquisition and analysis. Transient capacitance was compensated and output bandwidth was 3 kHz. Instantaneous and steady-state currents evoked by 5 mV pulses were monitored in experiments, which were applied to calculate series and input resistance. 10 µM TBOA (an antagonist of glutamate transports, TOCRIS) was added to the slices at the end of experiments to identify uGTCs, and 10 µM CNQX (6-Cyano-7-nitroquinoxaline-2,3-(1H,4H)-dione, SIGMA) was added to test GluR-mediated uEPSCs.

### A recording of spike patterns

The sequential spikes in GABAergic neurons were evoked by the signals integrated from the synapses in quantum release (uEPSC amplitudes in uniform) and those in non-quantum release with variable uEPSCs. These integrated signals were converted into ‘abf’ format for interface with Clampex. By an amplifier (MultiClamp-700B), the integrated currents were injected to evoke the sequential spikes that were inputted into pClamp-9 for data acquisition and analysis. Input resistance is balanced, and output bandwidth is 4 kHz.

### Standard pipette solution for whole-cell recordings

It contained (mM) 150 K-gluconate, 5 NaCl, 0.4 EGTA, 4 Mg-ATP, 0.5 Tris-GTP and 4 Na-phosphocreatine, 10 HEPES (pH 7.4 adjusted by 2M KOH). Fresh pipette solution was filtered with a 0.1 µm centrifuge filter before use. The osmolarity of pipette solution was 295–305 mOsmol, and the resistance of pipettes was 8–10 MΩ to have a good access and prevent run-down in synaptic responses [50].

### The analyses of uEPSCs and uGTCs

Electrical signals were acquired by a Digidata-1320A with pClamp-9. uEPSCs and uGTCs in response to stimuli 1∼5 were measured by Clampfit if the resting membrane potentials reached to −65 mV for the GABAergic neurons and −90 mV for the astrocytes. The data were analyzed if there were no significant changes in the resting membrane potentials, action potentials and series/input resistances throughout experiments. Indices in transmitter release patterns include the histograms of uEPSCs and uGTCs. Data for uEPSCs and uGTCs induced among multiple pulses were statistically compared by t-test. It is noteworthy that the responses of unitary synapses were analyzed before seeing the run-down in quantal sizes (usually 20∼25 min during the recordings), since the run-down in uEPSCs that is caused by the disturbance of intracellular environment may lead to the fluctuation of quantal sizes.

### The Computational integrations of synaptic inputs

The signals were integrated from numerous excitatory synapses and/or inhibitory ones. In the computational integrations of presynaptic excitatory inputs that are activated randomly, presynaptic cells (j = 1, 2, …J) fire spikes at a specific rate, which evoke synaptic currents (i, i.e., uEPSCs) in a postsynaptic neuron at time t_1_, t_2_, ……t_n_. The integrated input currents (I) can be described. 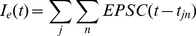
(1)


*t_jn_* represents the time of EPSC evoked by spike *n* at presynaptic neuron *j*. The integrated inputs are presumably correlated with uEPSCs, in which 
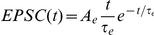
 [51], [52]. This is a simplified way to state the characteristics of low-pass filter in synaptic transmission in that currents are required to rise rapidly. In reality, the rising and decaying phases of synaptic currents are slowly developed, and the synapses are driven by multiple presynaptic spikes. Thus, we should apply the following kernel for presenting two sequential synaptic responses,
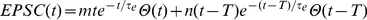
(2)in which *m* and *n* are the amplitudes of uEPSC one and two; and τ represents time constants. T is the time interval of inter-pulses at a synapse, and 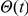 is Heaviside step function with 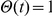 for t>0, and 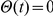 under other conditions.

Quantitative values used in the integration of currents from a population of glutamatergic unitary synapses on GABAergic neurons are listed below. 1) The firing rate (F) of presynaptic pyramidal neurons is 17Hz on average. 2) As asynchronously firing spikes in presynaptic neurons, inter-input intervals are 0.6∼1.6 ms. 3) uEPSC1∼5 amplitudes are 10.6±2.1 pA (Figs. 1∼2). Fluctuations in synaptic strength for constant vs. variable quantal size are 1 SD and 4SD, respectively. 4) The number of glutamatergic synapses on a postsynaptic neuron are presumably 250∼300. 5) The probability of releasing synaptic vesicles is a range of 0.2∼0.5. The integration was conducted by self-program in MatLab.

In the computational integrations of presynaptic inhibitory inputs that are activated randomly, presynaptic cells (*k* = 1, 2, …K) fire spikes at a specific rate, which evoke synaptic currents (i, i.e., uIPSCs) in a postsynaptic neuron at time t_1_, t_2_, ……t_n_. The integrated input currents (I) can be described.
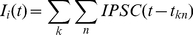
(3)


*t_kn_* represents the time of IPSC evoked by spike *n* at presynaptic neuron *k*. Quantitative values for GABAergic synapses on principal neurons include the followings: 1) Firing rate at presynaptic GABAergic neurons is about 30 Hz. 2) The number of inhibitory synapses on a postsynaptic pyramidal neuron is a range of 50∼75. 3) These GABAergic cells are asynchronous, i.e., inter-input intervals vary in a range of 0.6∼1.2 ms. 4) uIPSCs are 15±5 pA in amplitudes (our data not shown). 5) uIPSCs may drive precise encoding [53]. 6) The probability of releasing synaptic vesicles is in a range of 0.8∼1.0.

With these data, we integrated excitatory and inhibitory synaptic events including EPSCs and IPSCs, based on the following equation [51]. Here, the integrations of synaptic currents [I(t)] equal to the sum of EPSCs and IPSCs.
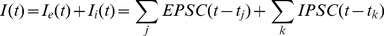
(4)


### Action potentials evoked by the integrations of synaptic inputs in simulation

The correlation between the integrated inputs from excitatory plus inhibitory synapses and the generation of action potentials is based on a principle, the integrate-and-fire neurons [54]. The currents of driving spike initiation are the mathematical addition from membrane leakage currents and integrated synaptic currents that are taken from equation 5. 
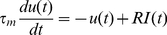
(5)


In this equation, τ presents membrane time constant, U(t) is membrane potentials, and R is membrane input resistance. Once the sum of synaptic currents [I(t)] drive the membrane potential to threshold potential (v in equation 6), action potentials are initiated, in which we introduced an active conductance to cell membrane. If input signals above threshold fall into the refractory periods of proceeding spikes, they are accounted to be not effective.
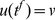
(6)


Quantitative values for glutamatergic and GABAergic synapses on a pyramidal neuron are taken from the data above. The resting membrane potentials and threshold potentials of pyramidal neurons are −65±2 mV and 22±1.5 mV, respectively [32].
